# Enhanced tumor penetration for efficient chemotherapy by a magnetothermally sensitive micelle combined with magnetic targeting and magnetic hyperthermia

**DOI:** 10.3389/fphar.2022.1045976

**Published:** 2022-11-18

**Authors:** Yu Wang, Rui Wang, Lixin Chen, Lili Chen, Yi Zheng, Yuanrong Xin, Xiqiu Zhou, Xiaoyun Song, Jinzhou Zheng

**Affiliations:** ^1^ Department of Surgery, PuDong Branch of Longhua Hospital, Shanghai University of Traditional Chinese Medicine, Shanghai, China; ^2^ School of Pharmacy, Jiangsu University, Zhenjiang, China

**Keywords:** thermosensitive polymer, magnetothermal-responsive drug release, magnetic targeting, magnetic hyperthermia, enhanced tumor penetration

## Abstract

The high accumulation and poor penetration of nanocarriers in tumor is a contradiction of nanomedicine, which reduces the efficacy of chemotherapy. Due to the positive effect of hyperthermia on *in vivo* drug diffusion, we designed a magnetothermally sensitive micelle (MTM) by integrating magnetic targeting (MT), magnetic hyperthermia (MH), and magnetothermally responsive drug release to facilitate simultaneous drug accumulation and penetration in tumor. Accordingly, we synthesized a cyanine7-modified thermosensitive polymer with phase transition at 42.3°C, and utilized it to prepare drug-loaded MTMs by encapsulating superparamagnetic MnFe_2_O_4_ nanoparticles and doxorubicin (DOX). The obtained DOX–MTM had not only high contents of DOX (9.1%) and MnFe_2_O_4_ (38.7%), but also some advantages such as superparamagnetism, high saturation magnetization, excellent magnetocaloric effect, and magnetothermal-dependent drug release. Therefore, DOX–MTM improved *in vitro* DOX cytotoxicity by enhancing DOX endocytosis under the assistance of MH. Furthermore, MT and MH enhanced *in vivo* DOX–MTM accumulation and DOX penetration in tumor, respectively, substantially inhibiting tumor growth (84%) with excellent biosafety. These results indicate the development of an optimized drug delivery system with MH and MH-dependent drug release, introducing a feasible strategy to enhance the application of nanomedicines in tumor chemotherapy.

## 1 Introduction

Nanosized drug delivery system can increase chemotherapeutic drug distribution in tumors by passive targeting strategy, which is an enhanced permeability and retention (EPR) effect (Maeda et al., 2000). Till date, various nanocarriers have been developed to simultaneously improve drug efficacy and reduce side effects through EPR-dependent drug accumulation in tumor (Guo et al., 2020; Zhang et al., 2021). Although EPR effect has been confirmed in the murine tumor model, the positive consequences of nanotechnology for clinical application are less clear (Harrington et al., 2001). Even in the murine model, EPR effect did not always positively influence the therapeutic efficacy of micelle drugs (Chen et al., 2018). Because of elevated interstitial fluid pressure (IFP) and high-density extracellular matrix of tumor tissue (Heldin et al., 2004), nanoparticles around 50–200 nm suitable for EPR effect are distributed preferentially around the tumor vasculature, instead of the tumor deep tissue (Minchinton and Tannock, 2006; Barua and Mitragotri, 2014). Moreover, an ideal drug delivery system should prolong drug blood circulation by stable encapsulation, until drug release in the tumor cells (Wang et al., 2016; Keller et al., 2020); this is because small drugs leaking from nanocarriers could be cleared rapidly by the kidney. Therefore, a great challenge still exists in optimizing targeted drug delivery system, which should overcome the dilemma between preferential distribution of larger nanoparticles (50–200 nm) in tumor tissue by EPR effect and penetration of very small nanoparticles (15–20 nm) (Perrault et al., 2009; Sykes et al., 2014) into the deep tumor tissue by simultaneous diffusion.

To address the inadequate distribution of nanocarrier-mediated chemotherapeutics in the deep tissue of tumor, some strategies including utilizing a combination of passive and active targeting, integrating passive targeting and shrinking nanocarrier particle size under tumor microenvironment, and enhancing nanocarrier penetration directly by modifying tissue-penetrating peptides, have been developed (Ruoslahti, 2012; Liu et al., 2015). Among these strategies, the combination of passive and active targeting was still limited by nanocarrier penetration, because active targeting-mediated endocytosis only occurred on the cellular surface after successful nanocarrier penetration into tumor tissue. Integrating passive targeting and shrinking nanocarrier particle size under tumor microenvironment depends on cleaving the stimuli-sensitive bonding to decrease nanocarrier size under the acidic microenvironment of tumor tissue. However, two issues in the strategy are nonnegligible. On one hand, the pH gap between tumor tissue (6.5–6.8) (Webb et al., 2011) and physiological environment (7.35–7.45) is insufficient for acid-sensitive cleavage (Xiong et al., 2020), reducing the efficiency of particle size shrinking. On the other hand, the size distributions of shrunken nanoparticles (30–50 nm) under acidic microenvironment are usually larger than 20 nm, which still hinders efficient penetration for drug delivery. With respect to enhancing nanocarrier penetration directly by modifying tissue-penetrating peptides, although previous studies have showed that tissue-penetrating peptides enhance efficient penetration of nanoparticles in tumor tissue by activating a complex and unclear endocytic transport pathway (Ruoslahti, 2017). Up to now, it is hard to design tissue-penetrating peptides with high transport efficiency for most types of tumors (Sharma et al., 2017).

Although nanocarrier diffusiveness through interstitial matrix of tumor may be inhibited by their large diameter, the low-molecular-weight chemotherapeutic drugs could easily spread in the tumor tissue by diffusion (Kaur et al., 2021). Therefore, specific nanocarrier-mediated chemotherapeutic drug accumulation in tumor vasculature and perivascular tissues, followed by rapid drug release and simultaneous accelerated drug penetration into deep tissue of tumor under a certain stimulation, is another promising approach. According to the positive effect of hyperthermia on drug diffusion (Cope et al., 1990; Schuster et al., 1995), a combination of thermosensitive nanocarrier and hyperthermia should be an ideal strategy, where the thermosensitive nanocarrier could protect the drug in the circulatory system and accumulate drug in tumor site, hyperthermia could efficiently trigger drug release and facilitate drug penetration. Therefore, thermosensitive liposomes have been utilized to drive doxorubicin (DOX) accumulation successfully into inside matrix of tumor in combination with local hyperthermia (Manzoor et al., 2012; Li et al., 2013). Although traditional thermosensitive liposomes have been used in the clinic, their thermo-sensitivity depended on gel-to-liquid transition of dipalmitoylphosphatidylcholine (DPPC) mainly at 41°C, which could only induce 40% drug release in 30 min at 42°C. Due to rapid blood flow, the nanocarrier could stay in tumor vasculature less than 2 min, the traditional thermosensitive liposome was not enough for rapid thermosensitive drug release (Gaber et al., 1995; Chen et al., 2008; Mo et al., 2019). Compared to lipid, thermosensitive polymers showed more sensitivity on temperature, as they could occur phase change promptly under certain temperature, inducing thermo-sensitive drug release efficiently. A typical thermosensitive polymer was poly(N-isopropylacrylamide) (pNIPAM), which presented high thermo-sensitivity around 32°C by themselves, with adjustable low critical solution temperature (LCST) varying from 32°C to 50°C (Qu et al., 2014b). Due to the excellent thermo-sensitivity of pNIPAM, it has been utilized to modify the thermo-sensitivity of traditional thermosensitive liposomes (Mo et al., 2019). Furthermore, the thermosensitive micelles based on poly(N-isopropylacrylamide) (pNIPAM) have been developed successfully (Cintra et al., 2022), which could trigger rapid drug release. As the result, pNIPAM-based thermosensitive micelles might serve as optimized candidates to combine with hyperthermia, thereby improving the distribution and therapeutic efficacy of chemotherapeutic drugs.

After selecting a suitable thermosensitive nanocarrier, non-invasive hyperthermia should also be considered carefully. Although near-infrared (NIR)-mediated photothermal treatment has been employed widely in this field, its drawbacks with respect to depth-limitation restrict its application in clinical studies (Stolik et al., 2000). Compared to photothermal treatment, superparamagnetic nanoparticle-induced magnetic hyperthermia (MH) has more potential for tumor treatment because of the high penetration of alternative magnetic field (AMF) in the human body. Moreover, superparamagnetic nanoparticles are used not only in MH, but also to guide nanocarrier distribution by magnetic targeting (MT) (Qu et al., 2022). Consequently, MH should be an optimized option in this field.

**FIGURE 1:**
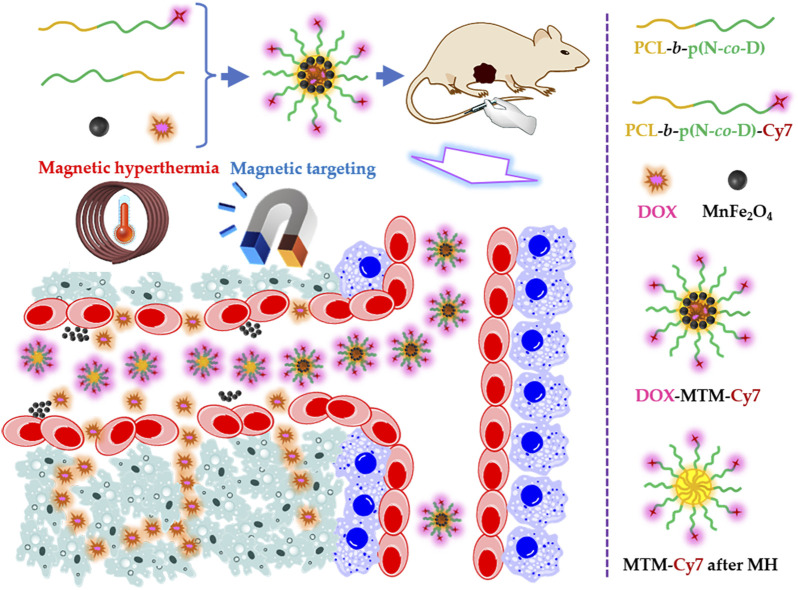
Schematic illustration of the doxorubicin (DOX)-loaded magnetothermally-sensitive micelle modified by Cy7 (DOX–MTM–Cy7) and its effect on enhancing DOX penetration under assistances of magnetic targeting and magnetic hyperthermia.

As reported in previous studies (Stolik et al., 2000; Manzoor et al., 2012; Li et al., 2013; Qu et al., 2014b; Mo et al., 2019; Qu et al., 2022), we designed magnetothermally sensitive micelles (MTMs), which integrate MT and MH into one versatile drug delivery system to enhance simultaneous drug accumulation and drug penetration in the tumor (Figure 1).

To fabricate MTM, we synthesized a thermosensitive polymer polycaprolactone-*b*-poly(N-isopropylacrylamide-*co*-N, N-dimethylacrylamide) [PCL-*b*-p(N-*co*-D)] with LCST around 42.3°C, and then modified it with cyanine 7 (Cy7) on its hydrophilic terminal, to synthesize PCL-*b*-p(N-*co*-D)–Cy7. Meanwhile, the monodispersed superparamagnetic nanoparticles MnFe_2_O_4_ were also synthesized to provide efficient MT and MH by high temperature organic phase decomposition. In addition, doxorubicin (DOX), a typical hydrophobic anti-tumor drug with fluorescence, was selected to identify drug endocytosis *in vitro* and tumor penetration *in vivo*.

We then fabricated drug-loaded MTMs by self-assembling these components under sonication. MTMs exhibited some advantages, such as suitable size for EPR effect, high saturation magnetization (*M*_s_), and excellent magnetocaloric effect and hypersensitive drug release under MH. Accordingly, they exhibited excellent anti-tumor potential *in vitro* and *in vivo*. *In vitro* cytotoxicity and cellular uptake of DOX were improved significantly by MH. After intravenous injection, MTM distribution in tumor could be enhanced by MT and identified by live Cy7 imaging. Moreover, under the effect of AMF, accumulated MTMs could generate local hyperthermia to induce rapid drug release from MTMs in tumor vessels and simultaneously enhance drug penetration, which was confirmed by DOX fluorescence. As drug accumulation in the tumor and distribution into the deep tumor tissue increased, the anti-tumor efficacy of MTM improved and side effects decreased under the assistance of MT and MH. These results suggest the development of a promising approach to overcome the dilemma of using nanomedicine, which suggested the application potential of magnetothermally sensitive nanocarriers in the clinic.

## 2 Materials and methods

### 2.1 Materials

ε-Caprolactone (ε-CL, 97%), stannous octoate [Sn (Oct)_2_, 92.5%–100%], N, N-dimethylacrylamide (DMAM, 99%), 1,2-hexadecanediol (97%), and oleylamine (>70%) were purchased from Sigma Aldrich (United States). N, N′-Dicyclohexylcarbodiimide (DCC, 98%), 4-dimethylaminopyridine (DMAP, 99%), and ethanolamine (EA, 95%) were purchased from Tokyo Chemical Industry (TCI, Japan). Iron (III) acetylacetonate [Fe(acac)_3_], manganese acetylacetonate [Mn(acac)_2_], benzyl ether (99%), and oleic acid (90%) were purchased from Alfa-Aesar. N-isopropylacrylamide (NIPAM), N, N′-azobisisobutyronitrile (AIBN), and triethylamine (TEA, 99%) were purchased from Aladdin (China). Benzyl alcohol (BaOH, 99%, Safe dry), anhydrous N, N-dimethyl sulfoxide (99.9%, DMSO), and anhydrous N, N-dimethylformamide (99.8%, DMF) were purchased from Admas. Dialysis tubing (8,000–14000 Da), dichloromethane (DCM), n-hexane, dioxane, and ethyl ether were purchased from Sinopharm Chemical Reagent Co., Ltd., (China). Sulfo-Cy7 maleimide (Sulfo-Cy7-Mal, 95%) was purchased from Kaixin Biotech. Co., Ltd., (China). DOX (97%) was purchased from Rhwan Chemical Reagent Co., Ltd., (China). 3-(4,5-dimethyl-thiazol-2-yl)-2,5-diphenyl tetrazoliumbromide (MTT) and 2-(4-amidinophenyl)-6-indolecarbamidine dihydrochloride (DAPI) were purchased from Beyotime Biotech Co., Ltd., (China).

AIBN was purified by recrystallization from ethyl alcohol. NIPAM was purified by recrystallization from n-hexane. DMAM was purified by reduced pressure distillation to remove monomethyl ether hydroquinone. DCM was desiccated by reflux with calcium hydride (CaH_2_) and purified by reduced pressure distillation. S-1-Dodecyl-S′-(α, α′-dimethyl-α″-acetic acid) trithiocarbonate (DDAT) was selected as chain transfer agent (CTA) for Reversible Addition–Fragmentation Chain Transfer (RAFT) polymerization based on literature (Lai et al., 2002). Monodisperse superparamagnetic nanoparticles (MnFe_2_O_4_) were synthesized as reported previously (Sun et al., 2004). Other reagents were used as received. The water used in all experiments was deionized using a Millipore Milli-Qsystem.

### 2.2 Synthesis of thermosensitive polymer

The thermosensitive polymer PCL-*b*-p(N-*co*-D) was synthesized as reported previously (Bian et al., 2012; Qu et al., 2014b) (Supplementary Figure S1). All reaction details are described below.

In the first step, polycaprolactone (PCL) was synthesized by ring-opening polymerization (ROP) by mixing 21.6 mg BaOH (0.2 mmol) as an initiator and 1,600 mg ε-CL (14 mmol) as monomers under anhydrous conditions with magnetic stirring. After adding Sn (Oct)_2_ as a catalyst, the reaction mixture was heated to 110°C for 12 h under argon (Ar_2_) atmosphere with magnetic stirring. At the end of the reaction, the product, PCL was purified three times by dissolving in DCM completely and successive precipitating in excess ethyl ether. The obtained product was dried finally in a vacuum oven at ambient temperature until the attainment of constant weight.

Then, a macro-CTA, PCL–DDAT, was prepared by esterification between PCL and DDAT, as reported previously (Bian et al., 2012; Qu et al., 2014b). Briefly, 1,260 mg PCL (0.2 mmol), 220 mg DDAT (0.6 mmol), 0.6 mmol DCC (124 mg), and 25 mg DMAP (0.2 mmol) were mixed and dissolved in DCM with magnetic stirring. Under nitrogen atmosphere and ambient temperature, esterification was carried out for 72 h. After the reaction, the precipitate was removed by filtration, while the collected supernatant was concentrated and precipitated by excess cold ethyl ether. To remove excess DDAT, the obtained precipitate was purified by reprecipitation in excess cold ethyl ether for four times. Finally, the purified PCL−DDAT was dried in a vacuum oven at ambient temperature and preserved in Ar_2_ under low temperature (−20°C).

Next, the thermosensitive polymer PCL-*b*-p(N-*co*-D) was synthesized using RAFT polymerization. All reagents, including 660 mg PCL–DDAT (0.1 mmol), 1,618 mg NIPAM (14.3 mmol), 565 mg DMAM (5.7 mmol), and 3.3 mg AIBN (0.02 mmol), were dissolved in 10 ml dioxane by magnetic stirring. Then, the mixture was thoroughly degassed by three freeze–pump–thaw cycles. The reaction was carried out at 70°C for 12 h under Ar_2_. The final product, PCL-*b*-p(N-*co*-D), was purified by repeated precipitation in excess cold ethyl ether and also preserved in Ar_2_ under low temperature (−20°C).

### 2.3 Fluorescence modification of thermosensitive polymer

As reported in previous studies (Nakayama and Okano, 2005; Akimoto et al., 2009), a facile, rapid, convenient one-pot reaction, involving simultaneous aminolysis of trithiocarbonate and thiol-ene click reaction, was employed in this study to modify the thermosensitive polymer with NIR fluorescence.

The obtained thermosensitive polymers (200 mg) were dissolved in 4 ml DMF, and then mixed with 10 mg sulfo-Cy7-Mal and 12 mg TEA (10 mol equivalents of terminal trithiocarbonate). The solution was degassed by three freeze–pump–thaw cycles. Meanwhile, 30 mg EA (40 mol equivalents of terminal trithiocarbonate) was dissolved in 1 ml DMF, deoxidized completely by Ar_2_ gas bubbling for 30 min, and injected into the mixed solution of thermosensitive polymers, sulfo-Cy7-Mal, and TEA. The obtained solution was deoxidized again rapidly *via* three freeze–pump–thaw cycles. Finally, the reaction was carried out at ambient temperature for 48 h under magnetic stirring and Ar_2_ atmosphere. Thereafter, the solution was dialyzed directly against water to remove the DMF, unreacted sulfo-Cy7-Mal, and excess EA and TEA. Finally, the purified PCL-*b*-p(N-*co*-D)-Cy7 was collected by lyophilization and preserved in the dark.

### 2.4 Characterization of thermosensitive polymer

The products of stepwise reactions were characterized by ^1^H NMR using CDCl_3_ or DMSO-d_6_ as the solvent, and identified by their chemical shifts relative to those of tetramethylsilane (TMS). The molecular weight (M_W_) and molecular weight distribution (M_WD_) of polymers were characterized by gel permeation chromatography (GPC, Agilent, PL-GPC220, England) using chloroform (CHCl_3_) as solvent. To characterize the thermo-sensitivity of PCL-*b*-p(N-*co*-D), its turbidity at high concentration (5 mg/ml) under varied temperatures (35°C–50°C) was measured at *λ* = 500 nm using an ultraviolet–visible (UV–Vis) spectrophotometer (Hitachi, U-3310, Japan) equipped with a temperature control unit. The LCST of PCL-*b*-p(N-*co*-D) was defined as the temperature producing a 50% decrease in optical transmittance (Chung et al., 1999). As reported previously (Qu et al., 2014b), the thermo-sensitivity of PCL-*b*-p(N-*co*-D) was also identified by dynamic light scattering (DLS, Malvern, Nano ZS90, United Kingdom), which measured size distribution of 0.5 mg/ml PCL-*b*-p(N-*co*-D) under varied temperatures. Moreover, the critical micellar concentration (CMC) of PCL-*b*-p(N-*co*-D) was characterized at 37 C by utilizing pyrene according to previous literature (Aguiar et al., 2003). The UV spectra of PCL-*b*-p(N-*co*-D), sulfo-Cy7-Mal, and PCL-*b*-p(N-*co*-D)-Cy7 were also measured using a UV–Vis spectrophotometer (Shimadzu, UV2600, Japan) with methanol as the solvent.

### 2.5 Preparation of doxorubicin-loaded thermosensitive micelles and doxorubicin-loaded magnetothermally sensitive micelle

All micelles were prepared by ultrasound-assisted self-assembly. For DOX–MTM preparation, all raw materials, including PCL-*b*-p(N-*co*-D), MnFe_2_O_4_, and DOX with 5/4/1 weight ratio, were completely dissolved in THF by oscillation. The mixed solution was then slowly added into excess deionized water (10×) under conditions of sonication, followed by dialysis against water for 48 h to remove THF and other impurities. The dialysis solution was purified by centrifugation (2,000 rpm, 10 min) to remove the sediment. Finally, DOX–MTMs were collected from the supernatant by lyophilization and stored at −20°C. DOX–MTM–Cy7 was also prepared similarly using PCL-*b*-p(N-*co*-D)-Cy7.

For DOX–MT preparation, a certain amount of PCL-*b*-p(N-*co*-D) and DOX with 9/1 weight ratio was adequately dispersed in THF. Then, the DOX–MTs were prepared using the same procedure. It was worth noting that all kinds of micelles containing DOX should be prepared and preserved in dark.

MTMs were also prepared using a similar procedure, with PCL-*b*-p(N-*co*-D) and MnFe_2_O_4_ with 1/1 weight ratio.

### 2.6 Characterization of doxorubicin-loaded micelles

The morphologies of DOX–MT and DOX–MTM were observed by high-resolution transmission electron microscopy (HRTEM, JEOL, JEM-2100, Japan). Their diameters were measured by DLS (Malvern, Nano ZS90, United Kingdom) at ambient temperature. DLS was also used in this study to determine the colloidal stability of DOX–MTM. After dispersing DOX–MTM in PBS with 0.5 mg/ml concentration, the time-dependent particle diameter was observed daily for 1 week.

The loading capacity and loading efficiency of DOX in DOX–MT and DOX–MTM were quantified using a fluorescence spectrophotometer (Shimadzu, RF-5301PC, Japan). The standard curve of DOX in a mixed solvent with THF and ethanol (1/9, w/w) was established at an excitation wavelength of 480 nm and an emission wavelength of 590 nm. Then the two DOX-loaded micelles were disassembled in THF with 1 mg/ml concentration and completely mixed with 9× ethanol. The DOX content in the DOX–MT mixed solvent was measured directly by fluorescence spectrophotometer, while the DOX–MTM mixed solvent should be purified by centrifugation (5,000 rpm, 10 min) to get rid of MnFe_2_O_4_. Then the DOX content in the supernatant was characterized as described.

The content of MnFe_2_O_4_ in DOX–MTM was measured by ICP-MS (Thermo Scientific, Xseries II, United States) to quantify the Mn and Fe contents. The magnetic properties of MnFe_2_O_4_ and DOX–MTM were characterized using a vibrating sample magnetometer (VSM, LakeShore 7404, United States) at 300 K. In addition, the magnetocaloric effect of DOX–MTM was evaluated by calculating its specific adsorption rate (SAR). DOX–MTM with 0.075 mg/ml[Mn + Fe] was dispersed into water, which was determined by ICP-MS. Then, 5 ml colloidal solution was placed in AMF generated by an AMF generator (SPG-20AB, ShuangPing Tech Ltd., China), with the AMF frequency (*f*) and strength (*H*_applied_) being 114 kHz and 63.6 kA/m, respectively. The inner diameter of the heating coil was 20 mm. The temperature change of the sample was recorded using a computer-attached fiber optic temperature sensor (FISO, FOT-M, Canada). Finally, the SAR was calculated using the formula described previously (Chang et al., 2018).

### 2.7 Drug release studies

The temperature-dependent drug release was investigated at 37°C and 43°C for 48°h, as described below. Briefly, 5 ml, 5 mg/ml DOX–MTM in PBS was dialyzed against 95 ml PBS in a shaking incubator for 48 h. At pre-determined time intervals, 1 ml solution was extracted from a dialysis bag outside and replaced with fresh PBS. The DOX content in the extracted solution was measured using a fluorescence spectrophotometer and the cumulative release was calculated.

Magnetothermal-responsive drug release from DOX–MTM was studied using a AMF generator (*f* = 114 kHz and *H*_applied_ = 63.6 kA/m). In this study, 2 ml 5 mg/ml DOX–MTM in PBS was dialyzed against 38 ml PBS under AMF for 10 min. During the time, 0.4 ml sample was extracted from outside the dialysis tubing every minute and measured using a fluorescence spectrophotometer to determine content of DOX. Simultaneously, an equal volume of corresponding buffer was added as a release medium.

The DOX release studies were repeated thrice. Therefore, the percentages of cumulative DOX release from the DOX–MTM under different conditions were determined finally by averaging three measurements.

To understand the relationship between DOX release and colloidal stability of DOX–MTM at 43°C and with additional MH, DOX–MTM particle diameters after corresponding treatment within 10 min were characterized by DLS. DOX–MTMs were dissolved in 40 ml PBS directly with 0.25 mg/ml constant concentration, which corresponded to the drug release study. A total of 2 ml DOX–MTM was extracted directly and detected by DLS immediately.

### 2.8 Cell experiment *in vitro*

4T1, a mouse-derived cell line mimicking stage IV human breast cancer, was purchased from the Chinese Academy of Sciences (Shanghai). It was cultured in RPMI 1640 medium (Hyclone) supplemented with 10% fetal bovine serum (FBS, Every Green, China) at 37°C in a humidified incubator with a 5% CO_2_ atmosphere.

*In vitro* cell viability was studied using a standard MTT assay, in which the biocompatibilities of PCL-*b*-p(N-*co*-D) and MTM were initially investigated. After confirming the high biocompatibilities of thermosensitive polymer and magnetic nanoparticle, the 4T1 proliferation was inhibited by different treatments, including DOX·HCl, DOX–MTM, and DOX–MTM + MH. For assessing the cytotoxicity of DOX·HCl and DOX–MTM, 1 × 10^4^ 4T1 cells in 100 μl corresponding medium were seeded in each well of 96-well plate. After incubation for 24 h under standard culture condition, the culture medium was replaced by medium containing DOX·HCl or DOX–MTM, where the final equivalent DOX concentration varied from 0.08 to 10 μg/ml. After incubating for 48 h, cell viabilities were investigated using the MTT assay.

Furthermore, for evaluating the cytotoxicity of DOX–MTM + MH, 2 × 10^5^ 4T1 cells in 2 ml corresponding medium were seeded in a 35 mm culture dish. After incubating for 24 h, the culture medium was replaced by medium containing 0.08–10 μg/ml DOX–MTM. To unify the concentration of (Mn + Fe) (75 μg/ml) in the study, MTMs containing 44.7% (w/w) MnFe_2_O_4_ were added to complement the shortage of (Mn + Fe) in the study. Then, the cells were exposed to AMF (10 min, 114 kHz and 15.9 kA/m) at the beginning of each 24 h and cultured under standard culture condition. At 48 h, cell viabilities were also quantified by MTT. As culture medium was selected as a negative control, cell survival rates after different treatments were calculated as the percentage of negative control values.

In addition to cytotoxicity, cellular uptake of DOX was also investigated using confocal laser scanning microscopy (CLSM, Leica, TCS SP5 II, Germany). Briefly, 2 × 10^6^ 4T1 cells were also seeded in a 35 mm culture dish. After incubating for 24 h, culture medium was replaced with 2 ml medium containing DOX–MTM with equivalent DOX content (2.5 μg/ml) and (Mn + Fe) concentration (75 μg/ml). For investigating the cellular uptake of DOX from DOX–MTM, 4T1 cells were cultured directly with DOX–MTM for 2 h. For investigating the cellular uptake of DOX from DOX–MTM + MH, 4T1 experienced MH of 10 min immediately under AMF after replacing the culture medium. Then, the cells were incubated under standard culture condition for prolonged 110 min. At the pre-determined time points, the culture medium was removed completely and the cells were washed thrice. Then, cells were fixed with 4% paraformaldehyde for 30 min at 4°C. After removing paraformaldehyde, the cells were stained using DAPI (2 μg/ml) for 10 min initially. After removing DAPI solution, the cells were washed with PBS thrice (10 min per time). Finally, the cells were sealed using glycerin and stored at 4°C, until observation using CLSM (excitation/emission: DAPI, 405 nm/455 nm; DOX, 488 nm/570 nm).

### 2.9 Animal protocol

Female Balb/c mice (4–5 weeks old, 18–20 g weight) were purchased from the laboratory animal center of Jiangsu University and housed in the animal center of Jiangsu University under specific pathogen-free conditions. Orthotopic breast tumors were established by injecting 4T1 cells (1 × 10^7^ cells per ml, 100 μl per mice) into the third or fourth mammary fat pads. After 1 week, tumors can grow to 80–100 mm^3^. The animals were treated in accordance with the ethical guidelines of Jiangsu University. The animal experiments were carried out in accordance with the regulations for animal experimentation issued by the State Committee of Science and Technology of the People’s Republic of China.

### 2.10 *In vivo* doxorubicin–magnetothermally sensitive micelle–cyanine 7 biodistribution and penetration

To facilitate the *in vivo* surveillance of DOX–MTM distribution using NIR imaging, PCL-*b*-p(N-*co*-D) was replaced with PCL-*b*-p(N-*co*-D)–Cy7 for preparing DOX–MTM–Cy7, which was fabricated using the same procedure described in Section 2.5.

When the tumors grew to 250–300 mm^3^, tumor-bearing mice were divided into two groups (*n* = 5), DOX–MTM–Cy7 alone and DOX–MTM–Cy7+MT. Then, DOX–MTM–Cy7 was intravenously injected into these mice with equivalent doses of DOX and Cy7 (5 and 1 mg/kg, respectively). For the DOX–MTM–Cy7+MT-treated mice, the button magnet with 0.18 T surface magnetic intensity (10 mm diameter and 4 mm thickness) was placed on the tumor area after injection immediately and maintained until NIR imaging. At pre-determined time points (4 and 20 h), the mice were anesthetized and imaged using a small animal *in vivo* imaging system (BRUKER, Xtreme II, Germany) equipped with an excitation bandpass filter at 720 nm and emission at 780 nm.

After the last NIR imaging, the mice injected with DOX–MTM–Cy7 for 20 h were treated with MH (20 min for each mouse, 114 kHz and 15.9 kA/m). One hour later, these mice were euthanized. The excised tumors were frozen sectioned at 10 μm thickness for immunofluorescence. Blood vessels were probed overnight using CD31 antibody (AF0099, Beyotime Biotech Co., Ltd., China) at 4°C, followed by incubation with FITC-conjugated secondary antibody (A0562, Beyotime Biotech Co., Ltd., China) for 2 h at room temperature. The nuclei were stained with DAPI. Finally, the tumor slices were observed using CLSM (DAPI, excitation/emission: 405 nm/455 nm; FITC, excitation/emission: 488 nm/520 nm; DOX, excitation/emission: 540 nm/580 nm).

### 2.11 *In vivo* tumor inhibition studies

When tumors grew to 80–100 mm^3^, these mice were divided into five groups (*n* = 6), followed by injection with PBS, free DOX·HCl, DOX–MTM, DOX–MTM + MT, and DOX–MTM + MT + MH, respectively. The DOX dose for each tumor-bearing mouse was limited at 5 mg/kg by injecting 0.1 ml solution at 1, 6, and 11 days. For DOX–MTM + MT- and DOX–MTM + MT + MH-treated mice, MT was performed after injection and maintained for 20 h. For DOX–MTM + MT + MH-treated mice, MH was operated under AMF (114 kHz and 15.9 kA/m) for 20 min after immediately removing the button magnet.

During the treatment, the tumor volume and body weight of tumor-bearing mice were measured and recorded at every 3 days, where tumor volume was calculated by following formula: V = AB^2^π/6, where A represents the larger diameter and B represents the smaller diameter. Tumor growth inhibition (TGI) was calculated as reported previously (Chandra et al., 2019).

### 2.12 Statistical analysis

All data were presented as mean ± standard deviation (SD). One-way analysis of variance (ANOVA) was used to determine significant differences between pairs of two groups. *p* < 0.05 and *p* < 0.01 were considered significant and extremely significant, respectively.

## 3 Results and discussion

### 3.1 Synthesis and characterization of thermosensitive polymer

The thermosensitive polymer was synthesized by a combination of ROP, esterification, and RAFT reactions (Supplementary Figure S1). PCL was selected as the hydrophobic segment of thermosensitive polymer because it is highly biocompatible and synthesized by ROP. Successful PCL synthesis was confirmed by H proton nuclear magnetic resonance (^1^H NMR; Supplementary Figure S2). Then, S-1-Dodecyl-S′-(α, α′-dimethyl-α″-acetic acid) trithiocarbonate (DDAT) was synthesized and its structure was also confirmed by ^1^H NMR (Supplementary Figure S3). PCL–DDAT, a macro-CTA, was prepared by esterification and characterized by ^1^H NMR (Supplementary Figure S4).

**FIGURE 2:**
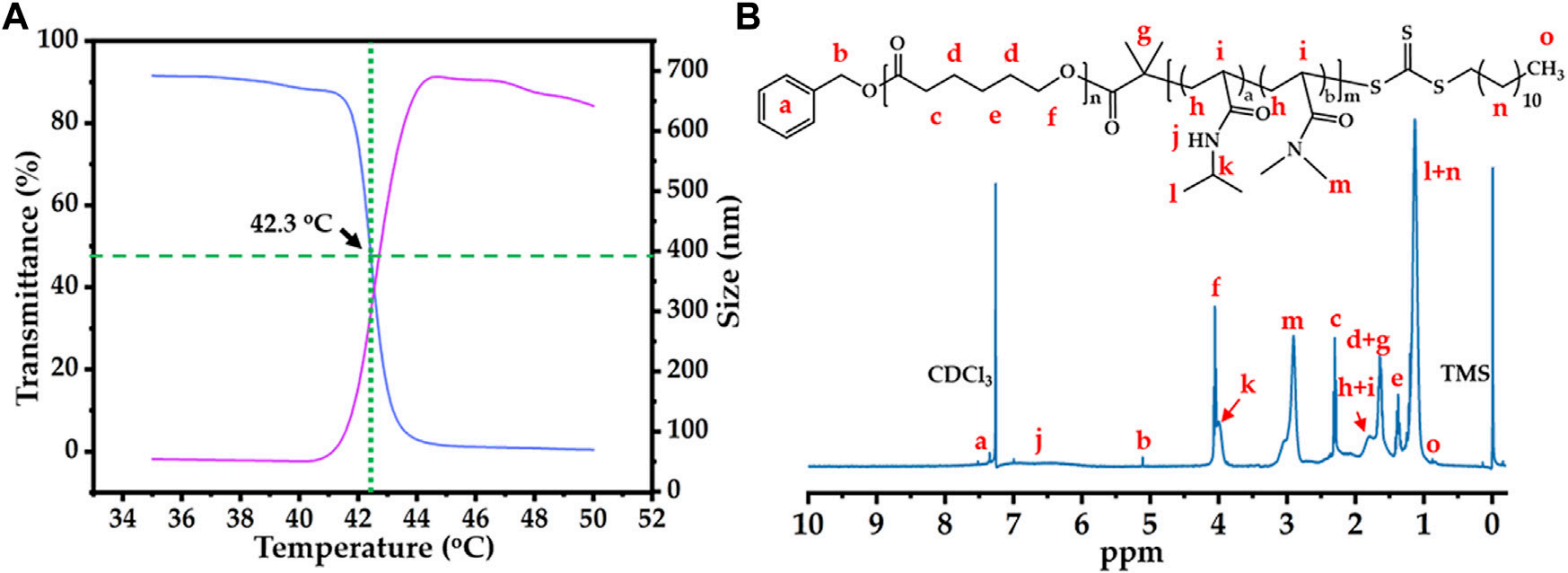
Thermo-sensitivity and structure of PCL-*b*-p(N-*co*-D): **(A)** temperature-dependent transmittance curve and diameter variation of PCL-*b*-p(N-*co*-D), low critical solution temperature (LCST): 42.3°C; **(B)** ^1^H NMR spectrum of PCL-*b*-poly(N-*co*-D), marking all characteristic peaks.

After successful macro-CTA synthesis, the thermosensitive polymer PCL-*b*-p(N-*co*-D) was synthesized by RAFT reaction, in which mixing units of NIPAM and DMAM were used to adjust the LCST of PCL-*b*-p(N-*co*-D). As reported previously (Qu et al., 2014b), the molar ratios of NIPAM/DMAM in the RAFT reaction were varied from 5/2.0 to 5/2.2, until the LCST of thermosensitive polymer was fixed around 42°C (Figure 2A). Moreover, its structure was characterized by ^1^H NMR (Figure 2B). Moreover, we selected the PCL-*b*-p(N-*co*-D) with suitable LCST to study its the M_W_ and M_WD_, meanwhile, the PCL was also studied as control. All results were showed in Supplementary Figure S5.

According to thermosensitive principle of pNIPAM (Schild, 1992), pNIPAM-based thermosensitive polymers should sharply transit from hydrophilicity to hydrophobicity in aqueous phase at the LCST, causing macroscopic phase separation. Therefore, the cloud point method, which quantifies the turbidity of thermosensitive polymers at 500 or 600 nm under different temperatures, is the simplest method for determining LCST of pNIPAM-based thermosensitive polymer. In this study, we researched the LCST of 5 mg/ml PCL-*b*-p(N-*co*-D) using cloud point under 35°C–50°C (Figure 2A, blue line). As PCL is a typical hydrophobic polymer, it usually precipitates in the aqueous phase. The high transmittance of PCL-*b*-p(N-*co*-D) below 41°C indicated that the thermosensitive segment, p(N-*co*-D), was polymerized successfully. As the temperature increased slightly, the light transmittance of PCL-*b*-p(N-*co*-D) decreased sharply, indicating the rapid phase transition of p(N-co-D), from hydrophilicity to hydrophobicity. When the temperature increased further (≥43°C), PCL-*b*-p(N-*co*-D) precipitated and the corresponding light transmittance was approximately 0%.

In addition to the cloud point method, a more sophisticated method, DLS, was employed for measuring the LCST of 0.5 mg/ml PCL-*b*-p(N-*co*-D) (Figure 2A, red line). The results indicated that the diameter of PCL-*b*-p(N-*co*-D) in aqueous phase decreased slightly from 54.5 nm (35°C) to 50.3 nm (41°C) as the surrounding temperature increased, indicating a coil-to-globule transition of pNIPAM at the temperature above its LCST. It is reasonable, because the thermosensitive segment of PCL-*b*-p(N-*co*-D), p(N-*co*-D), was a copolymer composed by two monomers, NIPAM and DMAM. As a typical hydrophilic monomer, DMAM disturbs the phase transition of pNIPAM at relatively high temperature (Qu et al., 2014b). When the surrounding temperature increased to 42°C, the diameter of PCL-*b*-p(N-*co*-D) increased sharply to 119.1 nm, indicating phase transition. PCL-*b*-p(N-*co*-D) diameter increased to 519 nm at 43°C because the agglomerate comprised a hydrophobic thermosensitive polymer. Then, the agglomerate size decreased slightly from 687 nm (45°C) to 642 nm (50°C), owing to molecular thermal motion. According to results shown in Figure 2A, we estimated the LCST of PCL-*b*-p(N-*co*-D) to be 42.3°C. Based on the result, PCL-*b*-p(N-*co*-D) should present morphology of micelle at the body temperature (37°C), we further investigated CMC of PCL-*b*-p(N-*co*-D) at 37°C. By contrasting fluorescence intensities of pyrene at 373 and 384 nm, the CMC of PCL-*b*-p(N-*co*-D) at 37°C was 2.7 mg/L, indicating its high stability at physiological environment.

After studying the LCST of PCL-*b*-p(N-*co*-D), we characterized PCL-*b*-p(N-*co*-D) structure using ^1^H NMR (Figure 2B). All characteristic PCL-*b*-p(N-*co*-D) peaks reported previously (Bian et al., 2012) were observed and marked, which confirmed the successful polymerization of p(N-*co*-D) as a product of PCL–DDAT chain propagation. Unfortunately, the average polymerization degrees of NIPAM and DMAM were hard to calculate by directly comparing the integral area ratios of relative peaks because the characteristic peaks of p(N-*co*-D) and PCL overlapped. However, the results clearly indicated that the thermosensitive polymer with suitable LCST was synthesized successfully, which could be utilized for the further study.

**FIGURE 3:**
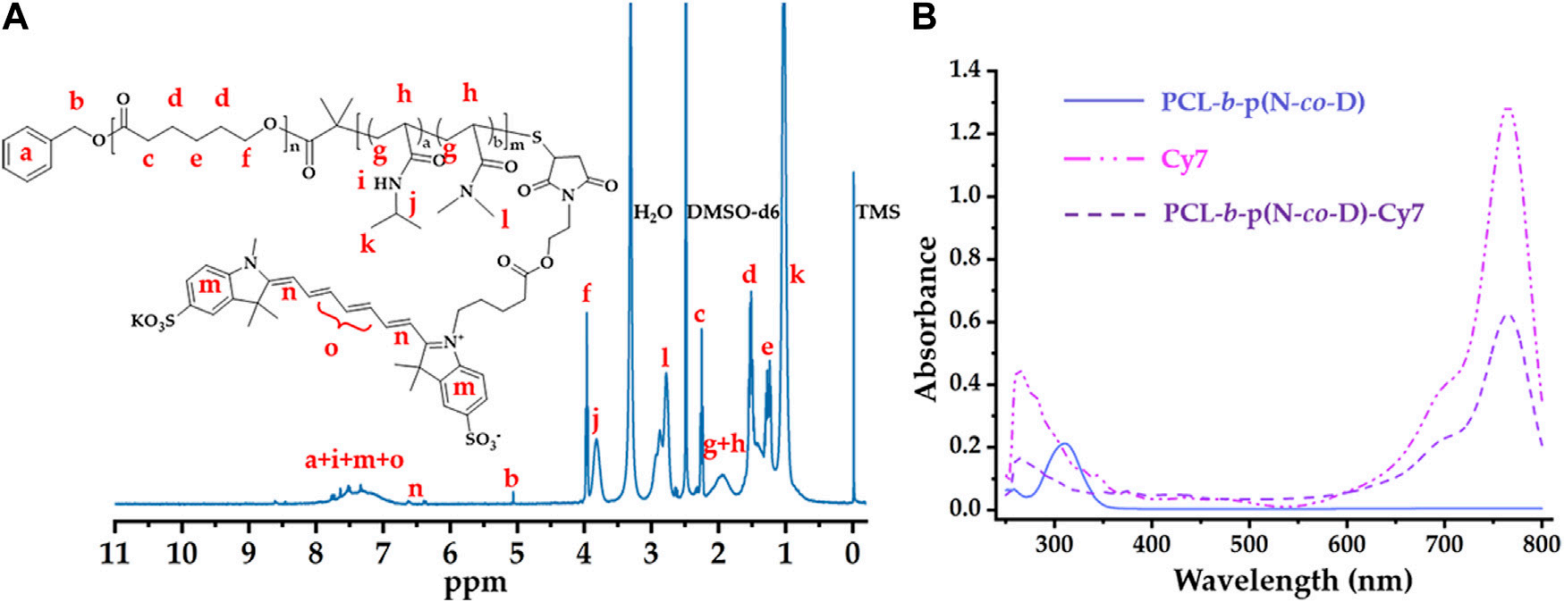
Structure characteristics of PCL-*b*-poly(N-*co*-D)–Cy7: **(A)** ^1^H NMR spectrum of PCL-*b*-poly(N-*co*-D)-Cy7, marking all characteristic peaks; **(B)** UV–Vis spectra of PCL-*b*-p(N-*co*-D), Cy7, and PCL-*b*-p(N-*co*-D)–Cy7.

After synthesis of PCL-*b*-p(N-*co*-D) with suitable LCST, it was further modified using Cy7 with sulfonic acid groups and maleimide; this is because sulfonic acid groups improved fluorescence of Cy7 in aqueous phase and maleimide improved the conjugation with PCL-*b*-p(N-*co*-D) trithiocarbonate. As trithiocarbonate end group of PCL-*b*-p(N-*co*-D) could be conserved into free thiol by aminolysis, which could efficiently react with maleimide. Consequently, the Cy7-labeled PCL-*b*-p(N-*co*-D) was synthesized by a combination of aminolysis and thiol-ene coupling reaction (Supplementary Figure S1). After the reaction, the product, PCL-*b*-p(N-*co*-D)-Cy7 was characterized by ultraviolet–visible (UV–Vis) spectrophotometry and ^1^H NMR (Figure 3).

Figure 3A shows the structure of PCL-*b*-p(N-*co*-D)-Cy7 and its corresponding ^1^H NMR spectrum in DMSO-d_6_. Compared with the ^1^H NMR spectrum in Figure 2B, characteristic terminal methyl proton peaks (at 0.88 ppm) were dispersed in this spectrum because of trithiocarbonate aminolysis. Moreover, as reported in previous studies (Bian et al., 2012; Zhang et al., 2019), all thermosensitive polymer and Cy7 characteristic peaks were identified (Figure 3A), suggesting successful PCL-*b*-p(N-*co*-D)–Cy7 synthesis.

Besides ^1^H NMR spectrum, UV–Vis spectra were employed to verify PCL-*b*-p(N-*co*-D) and PCL-*b*-p(N-*co*-D)–Cy7 structures. Apparently, PCL-*b*-p(N-*co*-D) exhibited a peak at 307 nm because of the presence of DDAT at terminal PCL-*b*-p(N-*co*-D) (Xu et al., 2010). However, the corresponding peak in PCL-*b*-p(N-*co*-D)-Cy7 curve dispersed because DDAT was removed from PCL-*b*-p(N-*co*-D) by aminolysis. Although the DDAT characteristic peak dispersed, PCL-*b*-p(N-*co*-D)–Cy7 showed all Cy7 characteristic peaks (Figure 3B). Furthermore, the Cy7 coupling efficiency with PCL-*b-*p(N-*co*-D) was approximately 100% according to the combination of the UV absorbance of Cy7 and PCL-*b*-p(N-*co*-D)-Cy7 at 765 nm, suggesting successful PCL-*b*-p(N-*co*-D) modification.

### 3.2 Characterization of doxorubicin-loaded magnetothermally sensitive micelles

**FIGURE 4:**
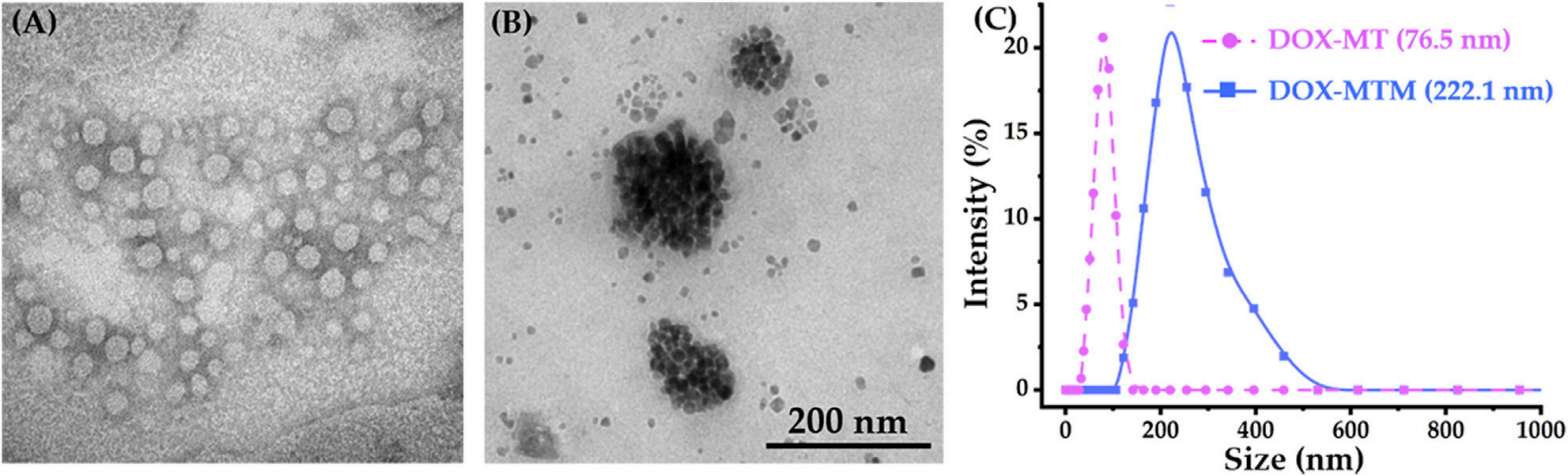
Morphologies (TEM) and particle size distributions (DLS) of DOX–MT and DOX–MTM: TEM result of **(A)** DOX–MTs and **(B)** DOX–MTMs; **(C)** DLS results of DOX–MT and DOX–MTM.

Using thermosensitive polymer PCL-*b*-p(N-*co*-D) with LCST at 42.3°C, we prepared DOX–MT and DOX–MTM by self-assembly. Then, their morphology and structure were observed directly by transmission electron microscopy (TEM) and DLS, respectively (Figure 4).

As PCL-*b*-p(N-*co*-D) is a typical amphiphilic macromolecule, it forms polymeric micelles by simple self-assembly and simultaneously encapsulates DOX. Owing to the surface tension of the micelle, DOX–MT had a spherical morphology according to phosphotungstic acid staining (Figure 4A), and the corresponding size was 76.5 nm (PDI = 0.149; Figure 4C). According to fluorescence standard curve of DOX in dimethyl sulfoxide (DMSO) at 593 nm, DOX–MT showed high loading capacity (9.4%, w/w) and loading efficiency (94%). Meanwhile, we prepared DOX–MTM to load MnFe_2_O_4_ and DOX simultaneously by same procedure. As MnFe_2_O_4_ nanoparticles showed high monodispersity in hexane (Supplementary Figure S5), amphiphilic PCL-*b*-p(N-*co*-D) could also encapsulate hydrophobic MnFe_2_O_4_ and DOX to easily form DOX–MTM (Figure 4B). Apparently, the particle size of DOX–MTM was bigger than that of DOX–MT, as it possesses a compact MnFe_2_O_4_ nanocluster core. DLS results also showed that the average diameter of DOX–MTM reached 222.1 nm (PDI = 0.197; Figure 4C), implying high MnFe_2_O_4_ content. Consequently, the MnFe_2_O_4_ and DOX contents in DOX–MTM were characterized by ICP-MS and fluorescence intensity at 593 nm, respectively. By conversion, DOX–MTM had high MnFe_2_O_4_ (38.7%, w/w) and DOX (9.1%, w/w) contents.

**FIGURE 5:**
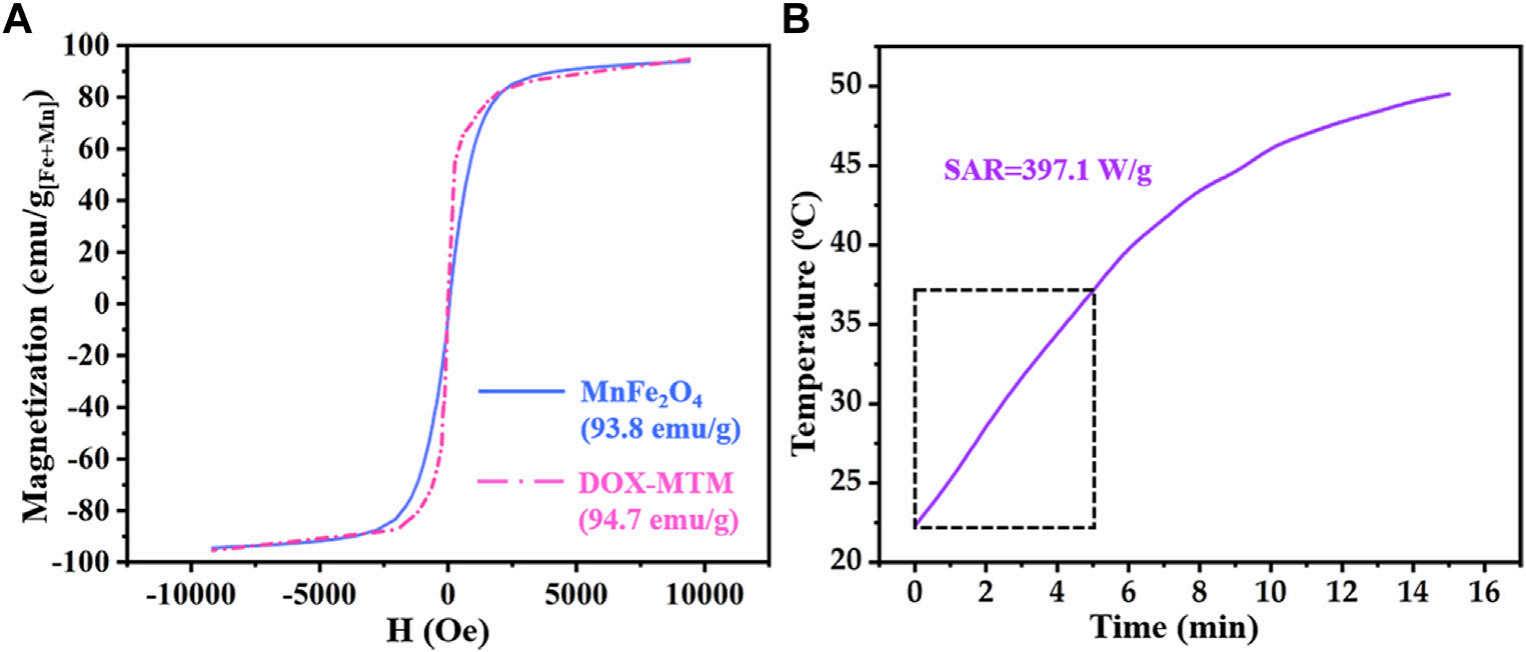
Magnetic property and magnetocaloric effect of DOX–MTM: **(A)** magnetization curves of MnFe_2_O_4_ and DOX–MTM at 300 K; **(B)** time-dependent temperature curve of DOX–MTM in AMF (frequency and strength as 114 kHz and 63.6 kA/m respectively) and corresponding SAR value.

To estimate DOX–MTM stability, the time-dependent hydrodynamic diameter was studied in PBS (Supplementary Figure S6), confirming their high stability in physiological buffer. Considering the suitable particle size for passive targeting (EPR effect, 50–300 nm), DOX–MTM had a potential on passive tumor targeting by EPR effect. Moreover, the high MnFe_2_O_4_ content also suggested the potential of DOX–MTM in MT and MH. Therefore, we further studied saturation magnetization (*M*_s_) and SAR of DOX–MTM (Figure 5).

According to relevant studies on monodispersed magnetic nanoparticles (Sun et al., 2004; Jang et al., 2009; Qu et al., 2014a), MnFe_2_O_4_ nanoparticles with diameter less than 10 nm showed superparamagnetism, which reappeared in Figure 5A. Interestingly, superparamagnetism is a critical property for the use of magnetic nanoparticles in medicine because it can decrease *in vivo* magnetic fluid aggregation. In addition, the *M*_s_ of MnFe_2_O_4_ nanoparticles was higher (93.8 emu/g_[Fe+Mn]_) than that of superparamagnetic Fe_3_O_4_ nanoparticles (Sun et al., 2004; Jang et al., 2009), facilitating its application in MT.

Due to the superparamagnetism of MnFe_2_O_4_ nanoparticles, DOX–MTM also showed the same superparamagnetism (Figure 5A). Although content of MnFe_2_O_4_ nanoparticles in DOX-MTM was 38.5%, the *M*_s_ of DOX–MTM also showed similar *M*_*s*_ (94.7 emu/g_[Fe+Mn]_). Considering the content of MnFe_2_O_4_ nanoparticles in DOX–MTM (38.7%, w/w), DOX–MTM also showed excellent magnetic property, which facilitated its application in medicine.

Besides high *M*_s_, DOX–MTM also exhibited substantial magnetocaloric effect and its heating profile and corresponding SAR are shown in Figure 5B. In a previous study (Qu et al., 2014a), the SAR value of DOX–MTM was calculated from the data of initial 5 min because a linear correlation was observed between time and temperature in the 5 min. However, the SAR reported in this study was lower than that reported previously (1,102.4 W/g) (Qu et al., 2014a). This is because this study used low strength AMF (*H*_applied_ = 62.8 kA/m), which was equivalent to 55.4% of that used previously. As low *H*_applied_ improves AMF biosafety and the SAR of magnetic fluid is proportional to the square of *H*_applied_ (Rosensweig, 2002), the low SAR value in the study is reasonable.

### 3.3 Drug release studies

**FIGURE 6:**
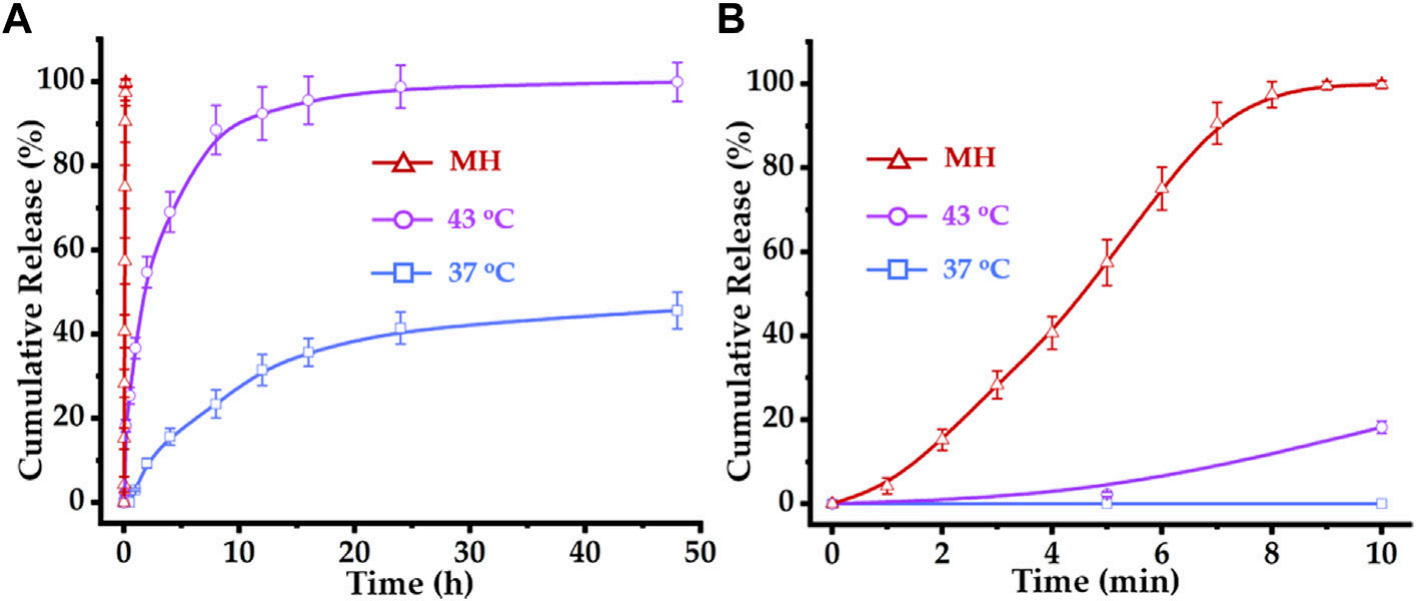
Drug release profiles of DOX–MTM at 37°C, 43°C and AMF (*f* = 114 kHz and *H*_*applied*_ = 63.6 kA/m): **(A)** long-term (48 h) and **(B)** short-term (10 min) drug release profiles of DOX–MTMs under corresponding conditions.

The thermosensitive micelles comprising PCL-*b*-p(N-*co*-D) lost their colloidal stability above their LCST (Figure 2A). Similarly, DOX–MTM comprising PCL-*b*-p(N-*co*-D) could be also destroyed at temperatures above 42.3°C, leading to drug rapid release. Therefore, DOX release from DOX–MTM was studied under different temperatures (37°C and 43°C) and MH (Figure 6).

Apparently, temperatures above PCL-*b*-p(N-*co*-D) LCST (42.3°C) slightly trigger the rapid DOX release from DOX–MTM. At 37°C, DOX was hardly released within 1 h. Then, DOX diffused slowly from DOX–MTM and the final cumulative release amount reached 41.5% within 24 h without further release. At 43°C, DOX–MTM released DOX continuously, as the cumulative DOX release reached 36.7% and 88.6% at 1 and 8 h, respectively. The cumulative DOX release increased to 98.9% at 24 h. However, it displayed an eruptible release behavior under the effect of MH as the cumulative DOX release curve under effect of MH was almost an upright line (Figure 6A). Since DOX release under MH was so rapid, its cumulative release reached 100% within 10 min.

To understand the relationship between DOX release and corresponding conditions (43°C and MH), we investigated the diameter of DOX–MTM under corresponding conditions by DLS (Supplementary Figure S7). DOX–MTM lost its colloid stability at 43°C because the temperature is slightly higher than PCL-*b*-p(N-*co*-D) LCST. Meanwhile, the DOX–MTM diameter could increase substantially under MH during its initial stage. Based on the thermo-sensitivity and magnetocaloric effect of DOX–MTM, AMF could efficiently heat the peripheral region of MnFe_2_O_4_ cluster, which is the corona of DOX–MTM composed of the thermosensitive hydrophilic p(N-*co*-D) segment. Therefore, MH could efficiently induce DOX–MTM phase transition and abruptly trigger DOX release, which corresponded to diameter change of DOX–MTM under AMF (Supplementary Figure S7).

Based on these results, we repeated the study under the same conditions for 10 min. The high SAR MnFe_2_O_4_ nanocluster efficiently triggered DOX release from DOX–MTM and the cumulative DOX release reached 100% almost within 9 min (Figure 6B). Meanwhile, incubation at 43°C triggered DOX release in the initial 10 min; however, cumulative DOX release at 43°C was substantially less than that at MH. Thus, this study provides visualized evidence for the high efficiency of MH on triggering drug release, which suggests the value of further MH use on controlled thermosensitive drug release *in vivo*.

### 3.4 *In vitro* anti-tumor efficacy

Cytotoxicity of the formulations were studied using 4T1 cells. Before evaluating the tumor inhibition ratio, PCL-*b*-p(N-*co*-D) and MTM biocompatibilities were investigated (Supplementary Figure S8). The cell survival rates after 0.2–2 mg/ml PCL-*b*-p(N-*co*-D) and MTM treatments approximated or even exceeded 100%, which indicated the excellent biocompatibility of amphiphilic thermosensitive polymer and polymer and MnFe_2_O_4_ composites.

**FIGURE 7:**
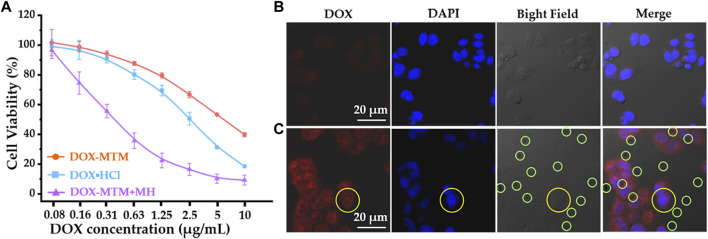
The cytotoxicity of 4T1 and cellular DOX uptake under different formulas: **(A)** cytotoxicity of 4T1 after treating with DOX·HCl, DOX–MTM, and DOX–MTM + MH (MH duration: 10 min, 114 kHz, and 15.9 kA/m); **(B)** cellular DOX uptake after DOX–MTM treatment for 2 h; **(C)** the cellular DOX–MTM + MH uptake (the same MH condition as cytotoxicity study) for 2 h.

Studies of *in vitro* anti-tumor efficacy (Figure 7A) revealed that treatment with different formulations for 48 h resulted in different 4T1 inhibition rates.

Free hydrophilic DOX (DOX·HCl) exhibited a higher inhibition rate than DOX–MTM under physiological conditions (Figure 7A). This is probably because DOX release from DOX–MTM under physiological conditions was relatively slow (Figure 6). Moreover, some studies have shown that the neutral hydrophilic membrane of nanocarrier, particularly poly(ethylene glycol) (PEG), inhibits the interaction between the nanocarrier and the negatively charged lipid envelope on the cell surface, resulting in less binding and low cellular uptake *via* endocytosis (Nakamura et al., 2012; Fang et al., 2017). Consequently, compared to DOX·HCl, the nanocarrier-protected drugs usually had low cytotoxicity *in vitro* (Xu et al., 2015; Ma et al., 2018; Xiong et al., 2020), which was verified in this study. Although DOX–MTM under physiological conditions exhibited relatively low cytotoxicity against 4T1, DOX–MTM + MH had the highest inhibition rate for the test period (Figure 7A). The inhibitory concentrations (IC_50_) of the three treatments, calculated from their cytotoxicity curves, were 5.36, 2.49, and 0.315 μg/ml for DOX–MTM alone, DOX·HCl, and DOX–MTM + MH, respectively. Thus, the cytotoxicity of DOX–MTM increased 17-fold by assistance of MH. Meanwhile, the cytotoxicity of MH was negligible, as cell viability after treatment with DOX–MTM + MH with the lowest DOX concentration (0.08 μg/ml) were comparable to those after treatment with DOX–MTM + MH with other DOX concentrations. Therefore, the high inhibition rate of DOX–MTM + MH was hard to attribute to the synergistic effect between hyperthermia and chemotherapeutic drug, which accompanied high MH-associated cell death reported in previous studies (Qu et al., 2014b; Tang et al., 2017; Ferjaoui et al., 2019).

Considering positive effect of MH on rapid DOX release from DOX–MTM (Figure 6), we further studied DOX cellular uptake by CLSM (Figures 7B,C).

Visually, after a 10 min exposure to AMF, intracellular DOX fluorescence intensity (red) increased after DOX–MTM + MH treatment for 2 h than that after DOX–MTM alone. The comparable results have been presented by previous studies (27,49), which have confirmed the efficiency of hyperthermia on facilitating drug endocytosis. Interestingly, intercellular hydrophobic drug usually distributed in cytoplasm after the effect of hyperthermia in these studies, which were insufficient for nucleus-targeted chemotherapeutic drugs, including DOX. In this study, we also observed similar phenomenon on DOX distribution, except one nucleus with DOX fluorescence (yellow circle; Figure 7C). Therefore, future studies should focus on nucleus-targeted drug delivery.

In addition, many dark spots are observed directly in the bright field and merged image (Figure 7C); meanwhile, their counterparts in Figure 7B show an individual dark spot. According to the previous studies, a dark spot is an aggregation of MTM after MH-induced phase transition. Theoretically, the normal MTM with colloid stability under physiological condition is hardly observed by optical microscope directly, because of its nanoscale diameter. After exposure to AMF for 10 min, MTMs would spontaneously agglomerate into large particles with micron-scale diameter (Supplementary Figure S8), leading to the phenomenon showed in Figure 7C.

### 3.5 *In vivo* tumor accumulation and penetration

**FIGURE 8:**
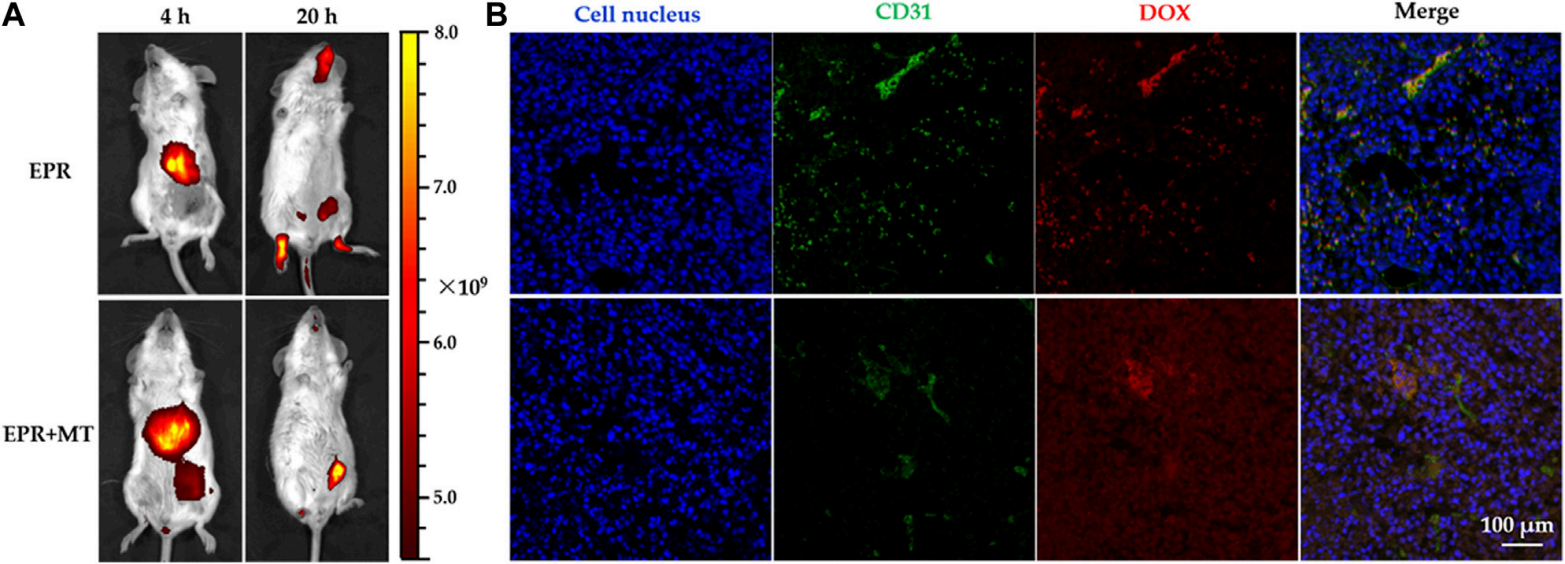
Near-infrared (NIR) *in vivo* imaging of tumor-bearing mice after injection of doxorubicin-loaded magnetothermally-sensitive micelle modified by Cy7 (DOX–MTM–Cy7) and DOX distribution in tumor after magnetic hyperthermia (MH) of 20 min: **(A)** Distribution of DOX–MTM–Cy7 in tumor-bearing mice after different targeting strategies (EPR or EPR + MT) 4 and 20 h post-injection; **(B)** the intra-tumoral DOX distribution of different targeting approaches after MH (20 min, 114 kHz and 15.9 kA/m) at 20 h post-injection, with cell nuclei staining (DAPI, blue), tumor vessels staining (FITC, green).

Due to the suitable diameter distribution, superparamagnetism, and high *M*_s_ of DOX–MTM, logically, it should possess a great potential on enhancing DOX accumulation in tumor by combining EPR and MT, as described previously (Schleich et al., 2014; Qu et al., 2022). Furthermore, if the combined targeting strategy was effective, the aggregate MnFe_2_O_4_ in tumor would generate sufficient energy under AMF to trigger rapid drug release and enhance drug penetration. Therefore, this study identified *in vivo* nanocarrier accumulation and successive drug penetration in tumor (Figure 8).

To monitor real-time *in vivo* distribution of DOX–MTM, we prepared Cy7-modified DOX–MTM (DOX–MTM–Cy7) by PCL-*b*-p(N-*co*-D)–Cy7, MnFe_2_O_4_, and DOX self-assembly, because Cy7 is a popular NIR dye used in *in vivo* non-invasive imaging. A strong fluorescent signal was detected in the liver 4 h after intravenous injection *via* the tail vein (Figure 8A), indicating that DOX–MTM–Cy7 primarily accumulates in the liver. This is an ordinary phenomenon because most non-PEGylated nanoparticles are rapidly sequestered by Kupffer cells residing in the liver sinusoids along with fenestrated endothelial cells (Wilhelm et al., 2016). However, the combined (EPR + MT) strategy influenced DOX–MTM–Cy7 accumulation in tumor more than pure EPR effect. Orthotopic breast cancer in the combined targeting was completely covered by Cy7 fluorescence (Figure 8A); meanwhile, non-fluorescence was detected at the tumor site after EPR. When the treatment time was prolonged to 20 h, most DOX–MTM–Cy7 was detected at the tumor site after EPR + MT. On the contrary, Cy7 fluorescence after EPR dispersed to different tissues, including muscles, tumor, and head. Although EPR effect has been well-documented in small animal models, it is not a solid targeting approach. As blood typically takes less than 1 minute to traverse the entire circulatory system in small animal model (Kaur et al., 2021), the duration of nanocarrier localization in tumor vasculature is very short. To improve EPR effect, MT was utilized to drive DOX–MTM–Cy7 distribution into tumor primarily in this study. Then, EPR effect acted as the second step to accumulate the nanocarrier by concentration-dependent extravasation. Therefore, the combined targeting strategy should efficiently increase nanocarrier accumulation.

However, the nanocarrier accumulation is not enough for tumor treatment. According to rapid proliferation of tumor cells, angiogenesis is a prerequisite for tumor growth. Compared to blood vessels of normal tissue, tumor vessels are highly irregular, convoluted and leaky vessels, which can reduce blood flow. Meanwhile, a high-density interstitial matrix composed of over-proliferative tumor cells can compress tumor vessels and block blood flow further. Therefore, most tumor tissue possesses increased IFP, which contributes to a decreased blood supply and transcapillary transport in tumors. As drug distribution by intravenous injection depends on transcapillary transport, high IFP is an obstacle to drug-treatment by decreasing concentration of therapeutic agents into tumor. As the result, we studied DOX distribution after MH, as shown in Figure 8B.

After live imaging, the mice were exposed to AMF for 20 min, then DOX distribution was studied by immunostaining of tumor tissue. Green and red fluorescence represented tumor vasculature and DOX distribution in Figure 8B, respectively. After MH for 20 min, the DOX fluorescence still overlapped with green fluorescence after EPR, indicating that DOX retention in tumor vasculature. On the contrary, after EPR + MT, DOX fluorescence permeated widely around tumor vasculature after (green fluorescence) and distributed deeply into tumor tissue after MH treatment. These interesting results reflected effective or ineffective MH. Logically, DOX–MTM–Cy7 accumulation after EPR effect was inadequate for generating effective hyperthermia under AMF, retaining DOX in DOX–MTM–Cy7. Due to the low penetration of DOX–MTM with large diameter (≥200 nm), DOX distribution was localized around tumor vasculature, which caused the overlapping of two fluorescence signals. On the contrary, under the effect of combined targeting strategy, a large amount of DOX–MTM–Cy7 were accumulated in tumor, corresponding to the live imaging, which could generate effective MH to trigger DOX release and enhance its diffusion.

### 3.6 *In vivo* anti-tumor efficacy

**FIGURE 9:**
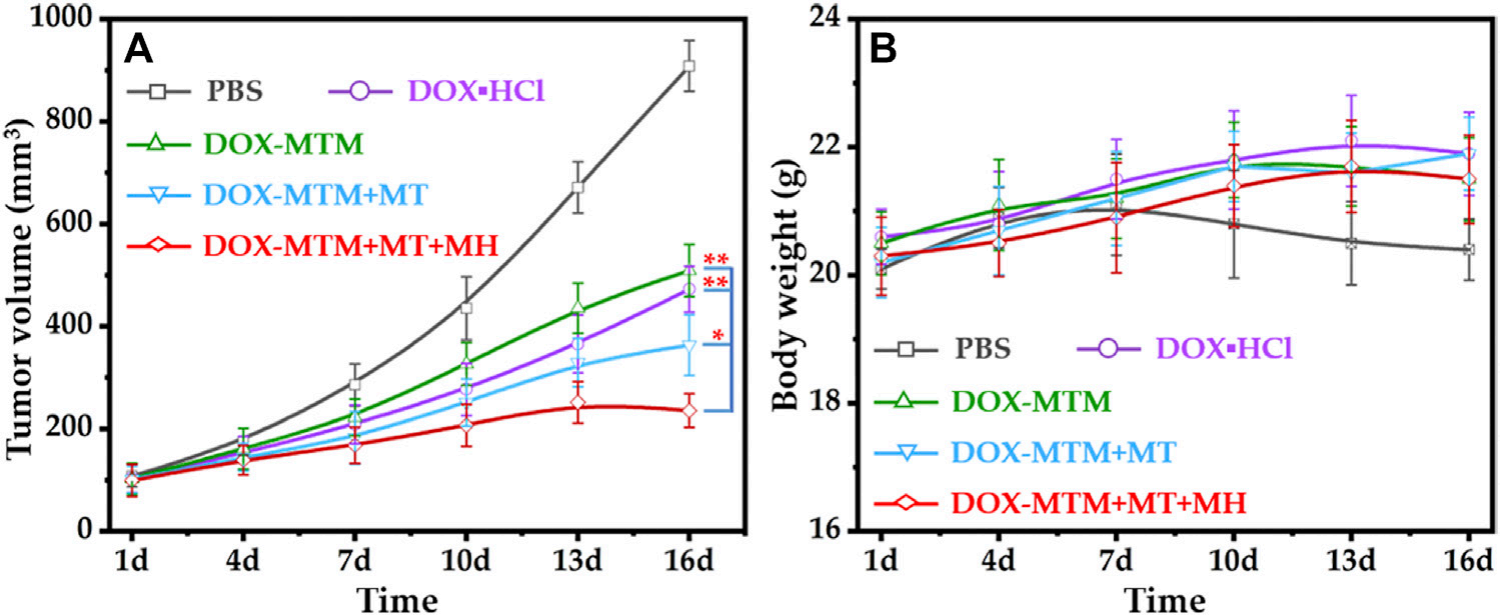
*In vivo* anti-tumor activities of different treatments: **(A)** the tumor volume curves after different treatments with the extension of curative time from 1 to 16 d (*p < 0.05; **p < 0.01); **(B)** the body weight curves after corresponding treatments with the extension of curative time from 1 to 16 d.

Due to the effects of DOX–MTM on *in vitro* increased 4T1 inhibition rate and *in vivo* enhanced tumor accumulation and drug penetration, we further investigated the *in vivo* anti-tumor efficacy of DOX–MTM (Figure 9).

Before performing the *in vivo* anti-tumor study, the local temperature of tumor under AMF should be explored. Unfortunately, the real-time temperature of tumor was hard to be monitored during MH because an overheated copper coil (≥50 °C) interfered with the measurement of infrared radiation (IR) thermal camera. Nevertheless, immunostained tumor sections indirectly indicated the effectiveness of *in vivo* MH. Therefore, in the *in vivo* anti-tumor study, we duplicated all parameters of MT and MH in DOX–MTM + MT- and DOX–MTM + MT + MH-treated mice.

The tumor volume changes during the treatment (1–16 d) indicated that all formulations with 5 mg/kg body weight equivalent DOX content could inhibit tumor growth than PBS (Figure 9A). Apparently, the tumor inhibition ratios of hydrophilic DOX·HCl and DOX–MTM alone were similar. The anti-tumor efficacy of DOX–MTM in combination with MT (DOX–MTM + MT) increased. Meanwhile, the anti-tumor efficacy of DOX–MTM in combination with both MT and MH (DOX–MTM + MT + MH) was enhanced further, as the tumor at all time points after DOX–MTM + MT + MH treatment were the smallest.

To quantify the anti-tumor efficacies of different treatments, the tumor growth inhibitions (TGIs) of different treatments were calculated at the end of the anti-tumor study, which were 54.4%, 49.8%, 68%, and 84% for DOX·HCl, DOX–MTM alone, DOX–MTM + MT, and DOX–MTM + MT + MH, respectively. Thus, the anti-tumor efficacy of DOX–MTM in combination with both MT and MH was significantly higher than that of other treatments. This is possible because the final anti-tumor efficacy of chemotherapeutic drug simultaneously depends on its accumulation and penetration. Although DOX–MTM could accumulate in the tumor by EPR effect theoretically, effectiveness of EPR alone was inadequate (Wilhelm et al., 2016). DOX–MTM accumulation increased after MT treatment (Figure 8A), which improved the *in vivo* anti-tumor efficacy of DOX–MTM. Furthermore, the accumulated DOX–MTM generated sufficient thermal energy to trigger drug release and enhance its penetration in the tumor (Figure 8B). Therefore, integrating enhanced accumulation by effective MT, efficient magnetothermally-responsive drug release, and effective *in situ* MH should be a promising strategy for a drug delivery system with high anti-tumor efficacy.

As DOX is extremely cytotoxic, the body weight of each mouse was measured to evaluate its safety (Figure 9B). To reduce cardiotoxicity of DOX, each formulation was injected at every 5 days. The long interval between two injections reduced the side effects of DOX, as indicated by the unfluctuating body weight of DOX·HCl-treated mice, which tended to increase during the treatment. The body weight of other formulation-treated mice also showed a similar tendency, as DOX–MTM is a drug delivery system with a stable diameter (Supplementary Figure S6) and slow drug diffusion under physiological condition. Moreover, the MH was biocompatible (Figure 9B), as the mice receiving MH also tended to gain body weight.

## 4 Conclusion

In summary, we present a promising strategy to resolve dilemma between the nanocarrier accumulation and small drug penetration by integrating MT, magnetothermally-responsive drug release and magnetothermally enhanced drug penetration into one composited micelle, i.e., MTM. By self-assembling the thermosensitive polymer, superparamagnetic MnFe_2_O_4_ nanoparticle, and DOX, the obtained DOX–MTM showed high contents of drug (9.1%) and MnFe_2_O_4_ (38.7%). Due to the high content of superparamagnetic nanoparticle and suitable LCST at 42.3°C, DOX–MTM exhibited excellent SAR and magnetothermally sensitive drug release; thus, the *in vitro* cytotoxicity increased to 17 times of itself and 7.9 times of DOX by magnetothermal-dependent DOX release and endocytosis. Moreover, DOX–MTM accumulation increased in the tumor under *in vivo* MT assistance because of superparamagnetism and high *M*_s_. Accordingly, subsequent MH triggered DOX release effectively and enhanced DOX penetration into deep tissue of tumor, which was confirmed by immunofluorescence of tumor tissue. Therefore, with the assistance of MT and MH, the DOX–MTM exhibited excellent TGI (84%) and high biosafety without any weight loss. This, we designed MTM, a promising delivery system with a great potential for clinical transformation, to solve the dilemma associated with nanocarrier-mediated chemotherapy.

## Data Availability

The original contributions presented in the study are included in the article/Supplementary Material, further inquiries can be directed to the corresponding authors.
